# Peripheral Nervous System Involvement in Sjögren’s Syndrome: Analysis of a Cohort From the Italian Research Group on Sjögren’s Syndrome

**DOI:** 10.3389/fimmu.2021.615656

**Published:** 2021-03-24

**Authors:** Giacomo Cafaro, Carlo Perricone, Francesco Carubbi, Chiara Baldini, Luca Quartuccio, Roberta Priori, Onorina Berardicurti, Francesco Ferro, Saviana Gandolfo, Angelica Gattamelata, Roberto Giacomelli, Salvatore De Vita, Roberto Gerli, Elena Bartoloni

**Affiliations:** ^1^ Rheumatology Unit, Department of Medicine, University of Perugia, Perugia, Italy; ^2^ Division of Rheumatology, Department of Biotechnological and Applied Clinical Science, University of L’Aquila, L’Aquila, Italy; ^3^ Rheumatology Unit, Department of Clinical and Experimental Medicine, University of Pisa, Pisa, Italy; ^4^ Department of Medical and Biological Sciences, Rheumatology Clinic, University of Udine, Udine, Italy; ^5^ Rheumatology Unit, Department of Internal Medicine and Medical Specialties, Sapienza University of Rome, Rome, Italy

**Keywords:** Sjögren’s syndrome, autoimmune diseases, autoantibodies, peripheral nervous system, sensorimotor polyneuropathy, pure sensory neuropathy

## Abstract

**Purpose:**

The prevalence of peripheral nervous system (PNS) involvement in primary Sjögren’s syndrome (pSS) has been reported to range from 2% to over 50%. Bias in study designs, including low number of patients and unclearly defined rheumatological and neurological diagnosis could explain such variability. Consequently, the exact depiction of PNS involvement in pSS is still lacking. This study aimed at analyzing the prevalence and the clinical and laboratory factors associated with PNS involvement in a very large cohort of well-characterized pSS patients with a clearly defined neurological diagnosis.

**Methods:**

Clinical and serological data of 1,695 pSS patients with specific and accurate information on PNS involvement were analyzed. Comparisons between patients with and without PNS involvement and between patients with distinct subsets of PNS manifestations were performed.

**Results:**

Prevalence of PNS involvement was 3.7%. The most frequent types observed were pure sensory neuropathies and axonal sensorimotor polyneuropathies (SMP). Patients with PNS involvement exhibited a more active disease profile and were more frequently treated with immunosuppressants. Intriguingly, clinical and serological negative prognostic factors, including purpura, extra-glandular manifestations, leukopenia, low complement and cryoglobulinemia, principally characterized patients with SMP, while subjects with pure sensory neuropathy displayed a milder phenotype.

**Conclusion:**

Our results highlight that PNS involvement is rather rare, but prognostically relevant in pSS. Main adverse prognostic features characterize patients with SMP, while pure sensory neuropathies are usually associated with a mild clinical picture. These findings, useful for patient stratification, may suggest protean pathogenic pathways underlying different types of PNS manifestations in pSS.

## Introduction

Primary Sjögren’s syndrome (pSS) is a systemic autoimmune disease mainly affecting exocrine glands. However, extra-glandular manifestations play a major role in the long-term prognosis of the disease (1, 2). Among these, neurological involvement represents a clinical challenge due to its heterogeneous presentation and diagnostic complexity (3–5). Although it may manifest in both central (C) and peripheral (P) nervous system (NS), CNS involvement has been commonly described as a rare complication of pSS, usually affecting a very low proportion of patients. On the contrary, PNS manifestations appear to be more frequent in pSS.

PNS has a wide spectrum of clinical phenotypes including axonal sensorimotor polyneuropathies (SMP), multiple mononeuropathies and cranial nerve neuropathies that have been described in pSS (3–5). However, it is intriguing to note that pure sensory neuropathies, in particular painful small-fiber neuropathy (SFN) and dorsal root ganglionopathy (DRG), seem to be peculiar features of the disease as they represent a considerable proportion of all PNS manifestations in pSS patients, and, conversely, pSS is one of the most frequent causes of pure sensory neuropathy among all immune-mediated diseases (6–8).

The true prevalence of PNS involvement in pSS is not clear and the reported frequency is extremely variable ranging from less than 2% to over 50% (3, 4). This impressive variability likely stems from three main causes: i) methodological differences in the approach to neurological diagnosis. In some studies all patients have been systematically screened for PNS disease, independently of the clinical picture, thus including a large proportion of asymptomatic subjects. ii) different settings of patient enrollment. Studies in which subjects are enrolled in a neurological setting represent a sub-group of patients with a higher probability of NS involvement, as well as cohorts enrolled among inpatients include more severe cases with a higher prevalence of extra-glandular manifestations of the disease, thus introducing a significant selection bias (9), iii) heterogeneity in the characteristics of study design. The cohorts of patients previously analyzed show a large variability of anti-Ro/SSA and anti-La/SSB status. Some do not systematically exclude patients with significant comorbidities, such as other concomitant CTDs or conditions that may contribute to PNS manifestations. Additionally, a significant proportion of currently available studies include patients diagnosed with pSS, though with both negative salivary gland biopsy and circulating specific autoantibodies (i.e. anti-Ro/SSA/anti-La/SSB) (4). Cohort sizes are also variable and, interestingly, the smaller cohort studies appear to be those with higher PNS involvement prevalence.

In order to overcome these limitations that significantly influence the findings in terms of prevalence of PNS involvement, its clinical impact and the serological and clinical features that may distinguish these pSS patients from those without PNS disease, we decided to analyze a very wide patients cohort recruited by the Italian Research Group on SS (GRISS), applying a real-life approach. The objective of this study was to characterize the epidemiological, clinical and laboratory features of pSS patients according to the presence of well characterized PNS involvement.

## Methods

**Figure 1:**
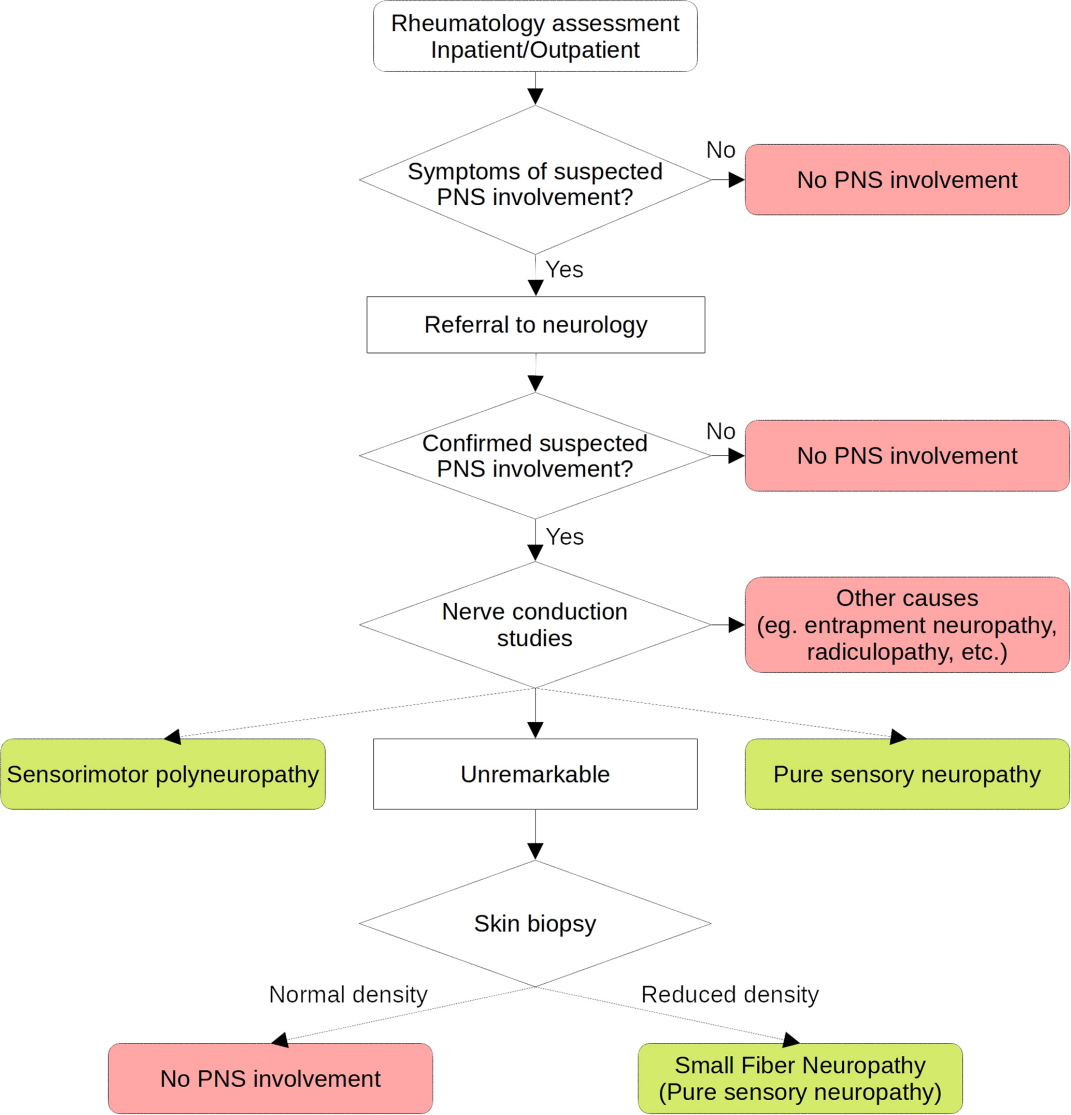
Flow-chart of the standardized protocol applied to classify peripheral nervous system involvement in the cohort.

This study was performed in line with the principles of the Declaration of Helsinki. Approval was granted by the Ethics Committee “Comitato Etico Regionale Umbria” (3780/19). Patients included in this retrospective cohort were enrolled by the five Italian centers of the GRISS (Perugia, Udine, Roma, L’Aquila and Pisa). This cohort includes both in and outpatients and the methodology applied to identify and classify patients was carefully homogenized following multiple meetings, in order to obtain a homogeneous population that carefully represents a real life cohort of pSS patients. Because a proportion of patients were included before the publication of 2012 and 2016 classification criteria, we applied those of the 2002 American-European Consensus Group (AECG) (9). As previously mentioned, in order to overcome most limitations of previous studies, common criteria were applied at enrollment. Clinical data included age at diagnosis, age at enrollment, history of extra-glandular manifestations (history of any extra-glandular clinical domain included in the EULAR Sjögren’s syndrome disease activity index (ESSDAI) (10), with the exclusion of PNS), Raynaud’s phenomenon. Because of their clinical impact in terms of lymphoproliferative risk, purpura, recurrent parotid swelling and history of lymphoma were recorded independently. Disease-specific laboratory markers included cytopenia, low C3 and C4 complement levels, hypergammaglobulinemia, rheumatoid factor, antinuclear antibodies (ANA), anti-Ro/SSA, anti-La/SSB antibodies and cryoglobulins, defined according to the relative laboratory cut-offs, ANA as ≥ 1:160. Ongoing and previous therapies recorded included glucocorticoids (GCs) (prednisolone ≥ 7.5 mg/day or equivalent), immunosuppressants (ISs) (azathioprine, cyclophosphamide, cyclosporine A, mycophenolate mofetil, methotrexate, leflunomide and rituximab) and hydroxychloroquine. Subjects with symptoms or clinical findings suggesting possible PNS involvement underwent physical examination performed by a neurologist and nerve conduction studies when suggested. Thus, subjects were classified as having SMP or pure-sensory neuropathy according to the results of nerve conduction studies. Patients with normal nerve conduction studies underwent skin biopsy in order to rule out SFN, according to a standardized technique (6). No quantitative sensory testing was performed prior to biopsy. No testing was performed on asymptomatic subjects and patients with other CTDs were excluded. Patients with peripheral neuropathies potentially determined by other causes, including paraneoplastic syndromes, alcoholism, B12 vitamin deficiency, diabetes mellitus, pharmacotherapy, systemic vasculitis not related to pSS, amyloidosis, paraproteinemia and focal entrapments, were excluded from the analysis. A flowchart describing the standardized protocol applied to classify patients according to PNS involvement is shown in **Figure 1**.

### Statistical Analysis

The chi-squared test and the Mann–Whitney *U*-test were used for comparisons of categorical variables and continuous variables, respectively. In order to account for type 1 error, Bonferroni correction was applied and data were considered significant for p ≤ 0.0025. Significant variables at univariate analysis were included in a multivariate logistic regression model.

## Results

The analyzed study cohort included 1,695 patients (4.6% males) with a median (range) age at diagnosis of 53 (10-86) years and median disease duration from diagnosis of 4 (0-45) years.

Raynaud’s phenomenon was the most prevalent extra-glandular manifestation. One third of patients reported a history of parotid swelling and nearly half were characterized by systemic extra-glandular involvement. Approximately one-third of patients were characterized by leukopenia. The most prevalent immunological features were ANA and anti-Ro/SSA positivity. Finally, 27% of patients had been treated or were receiving treatment with symptomatic therapy alone, 41% were on GCs and 18% of patients required treatment with IS agents, with 12% of patients taking both GCs and ISs.

The global prevalence of symptomatic PNS involvement was 3.7% (62/1695). In particular, 35 subjects presented a SMP and 25 a pure sensory neuropathy, of which 6 patients with SFN; two additional patients had a cranial nerve neuropathy and a mononeuritis multiplex, respectively.

When compared with patients without symptomatic PNS involvement, pSS subjects with evidence of PNS manifestations had a higher frequency of purpura, other organ involvement, low complement levels (C4) and cryoglobulinemia (**Table 1**). In addition, glucocorticoids and immunosuppressive drugs were more frequently used in these patients. At multivariate analysis adjusted for all significant variables at univariate analysis, higher risk of PNS involvement was independently associated with a multisystemic organ involvement (OR=64.0; 95% CI 8.8-467.5; p ≤ 0.0001), the presence of cryoglobulinemia (OR=3,5; 95% CI 1.8-6.7; p ≤ 0.0001) and the use of immunosuppressive treatments (OR=3.17 95% CI 1.8-5.5; p ≤ 0.0001). There was only a trend, but not statistically significant, toward an association with low C4 levels (OR=2.38; 95% CI 0.9-6.2; p ≤ 0.076).

**Table 1:** Demographic, clinical and immunological features of pSS patients with and without PNS involvement.

	No PNS involvement(N. 1633)	With PNS involvement(N. 62)	p value
Gender (male)	4.4%	8.1%	0.196
Age at diagnosis, median (range), years	53 (10-86)	51 (21-80)	0.570
Disease duration, median (range), years	4 (0-45)	5 (0-32)	0.019
Parotid swelling	29.4%	24.2%	0.458
Purpura	7.3%	23.0%	**0.0001**
Raynaud’s phenomenon	22.4%	29.0%	0.286
Other organ involvement	37.8%	98.4%	**0.0001**
Lymphoma	4.2%	8.1%	0.187
Leukopenia	24.2%	37.1%	0.03
Hypocomplementemia	19.2%	40.3%	**0.0001**
Low C3	16.4%	24.1%	0.169
Low C4	10.5%	31.0%	**0.0001**
Hypergammaglobulinemia	43.7%	43.6%	1.000
Monoclonal gammopathy	6.7%	14.5%	0.033
Antinuclear antibodies	87.0%	93.4%	0.171
Anti-SSA/Ro	66.1%	72.6%	0.359
Anti-SSB/La	32.2%	38.7%	0.350
Rheumatoid factor	45.8%	46.8%	0.984
Cryoglobulins	3.8%	19.4%	**0.0001**
Other autoantibodies	16.3%	31.2%	0.004
Glucocorticoid use	39.8%	73.7%	**0.0001**
Immunosuppressants	16.6%	54.4%	**0.0001**
Hydroxychloroquine	46.4%	46.0%	1.000

Significant results, as described in the Methods, are shown in bold.

We were then interested to verify possible demographic, clinical and immunologic differences according to the type of PNS manifestation. For this purpose, patients were subdivided according to the diagnosis of SMP or pure sensory neuropathy, the two main types of PNS manifestations we found in this study. Among the 34 patients with known onset time of SMP involvement, neurological diagnosis was made before pSS diagnosis in 3 (9%), at the same time in 10 (29%) and after in 21 (62%) patients, while the diagnosis of pure sensory neuropathy was antecedent in 3 (12%), simultaneous in 9 (36%) and subsequent in 13 (52%), to the diagnosis of pSS. In comparison to patients without PNS manifestations (**Table 2**), the SMP subgroup was clinically characterized by higher prevalence of purpura, more diffuse disease due to involvement of other organs, higher use of both glucocorticoids and immunosuppressive drugs and higher frequency of immunological biomarkers, such as leukopenia, low complement levels, cryoglobulins and additional autoantibodies. On the other hand, patients with pure sensory neuropathy were characterized only by a higher prevalence of extra-glandular involvement and more frequent use of immunosuppressive drugs with respect to patient without PNS involvement, while no difference was found for other markers of disease severity.

**Table 2:** Demographic, clinical and serological features of pSS patients with sensory and sensorimotor neuropathy in comparison to patients without PNS involvement.

	No PNS involvement (N. 1633)	Pure sensory neuropathy (N. 25)	p value	Sensorimotor polineuropathy (N. 35)	p value
Gender (male)	4.4%	4.0%	1.000	11.4%	0.069
Age at diagnosis, median (range), years	53 (10-86)	49 (21-80)	0.959	52 (38-79)	0.339
Disease duration, median (range), years	4 (0-45)	8 (0-26)	0.017	4 (0-32)	0.373
Parotid swelling	29.4%	8.0%	0.024	34.3%	0.659
Purpura	7.3%	16.0%	0.110	25.7%	**0.0001**
Raynaud phenomenon	22.4%	32.0%	0.370	28.6%	0.510
Other organ involvement	37.8%	100%	**0.0001**	97.1%	**0.0001**
Lymphoma	4.2%	8.0%	0.285	5.7%	0.656
Leukopenia	24.2%	20.0%	0.815	48.6%	**0.002**
Hypocomplementemia	19.2%	32.0%	0.175	45.7%	**0.0001**
Low C3	16.4%	21.7%	0.568	26.5%	0.184
Low C4	10.5%	21.7%	0.090	35.3%	**0.0001**
Hypergammaglobulinemia	43.7%	44.0%	1.000	45.7%	0.950
Monoclonal gammopathy	6.7%	16.0%	0.085	11.4%	0.293
Antinuclear antibodies	87.0%	100%	0.064	88.6%	1.000
Anti-SSA/Ro	66.1%	80.0%	0.200	68.6%	0.904
Anti-SSB/La	32.2%	44.0%	0.301	34.3%	0.939
Rheumatoid factor	45.8%	40.0%	0.707	51.4%	0.625
Cryoglobulins	3.8%	12.0%	0.072	22.9%	**0.0001**
Other autoantibodies	16.27%	24.0%	0.445	37.1%	**0.002**
Glucocorticoids	39.8%	60.9%	0.067	81.8%	**0.0001**
Immunosuppressants	16.6%	47.8%	**0.0001**	57.6%	**0.0001**
Hydroxychloroquine	46.4%	56.5%	0.440	45.5%	1.000

Significant results, as described in the Methods, are shown in bold.

## Discussion

The real prevalence of PNS manifestations in pSS patients is a matter of debate, as it has been reported with very disparate frequencies in different studies. According to the common current clinical practice, the impressive high proportion of pSS patients with PNS manifestations described in a number of previously published reports, up to 50%, appears to be overestimated with respect to that currently observed in rheumatological clinical practice (3, 5, 7). This overestimation derives mainly from studies including small cohorts with a patient selection bias recruiting without a definite rheumatological or neurological diagnosis.

We herein focused on symptomatic PNS involvement. This approach was chosen in order to identify a cohort of patients as close as possible to a real life rheumatological population. The frequency of symptomatic PNS involvement found in our study (3.7%) appears to be consistent with the range (1.8%-16%) reported in the most recent and reliable case series enrolling more than 250 pSS patients (8, 11–17) and, therefore, more comparable with our data (**Table 3**). To our knowledge, this is the largest study analyzing a well characterized cohort of pSS patients with definite PNS manifestations confirmed by specific instrumental diagnostic tools.

**Table 3:** Comparison of the main available studies investigating PNS involvement in pSS.

Author, yr (ref.)	SS criteria	Pts(N.)	Prevalence of PNS involvement	Prevalent type of PNS involvement	Anti-Ro/SSA^+^ in total pts. cohort	Anti-Ro/SSA^+^ in pts. with PNS involvement
Skopouli (8)	ECSG 1993	261	2.3%	Not defined	53%	NR
Garcìa-Carrasco (11)	ECSG 1993	400	7.0%	Not defined	40%	NR
Ramos Casals (12)	ECSG 1993	1010	11%	Not defined	52%	NR
Pavlakis (13)	AECG 2002	509	1.8%	Axonal 100% (sensory/SMP)	NR	89%
Brito-Zerón (14)	ECSG 1993	563	10%	SMP 44% Sensory 27% MNM 27%	NR	52%
Jamilluoux (15)	AECG 2002	420	15%	SMP 38% Sensory 43%	46%	32%
Carvajal Alegria (16)	AECG 2002	395	16%	SMP 28% Sensory 49%	58%	48%
Ye (17)	AECG 2002	415	16.6%	Not defined (only symptoms)	NR	NR
Present study	AECG 2002	1695	3.7%	SMP 56% Sensory 40%	66%	73%

ECSG, European Community Study Group; AECG, American-European Consensus Group; SMP, sensorimotor polyneuropathy; MNM, mononeuritis multiplex.

A relevant observation deriving from the analysis of similar previously published studies is that the pSS classification criteria adopted are not homogeneous and, more importantly, the proportion of patients with circulating anti-Ro/SSA antibodies, which represent markers of autoimmune disease and key elements for the diagnosis and prognosis definition of pSS, is rather low compared to that characterizing our cohort and other published studies (18). Similarly to the other studies (**Table 3**), we found that the two most frequent types of PNS involvement in pSS were SMP and pure sensory neuropathy. Interestingly, when compared with pSS patients without PNS manifestations, the subgroup with evidence of PNS involvement had a number of clinical and serological elements characterizing more diffuse and aggressive autoimmune disease (**Table 1**). However, our data also showed that these negative prognostic factors, mainly linked to increased lymphoprolipherative risk (purpura, hypocomplementemia and cryoglobulinaemia) essentially characterized the group of patients with SMP, while pSS subjects with sensory neuropathy presented a milder clinical and immunological profile (**Table 2**). In particular, these findings appear to fit with the evidence that the majority of pSS patients with SMP display more active and severe systemic disease defined by the presence of cutaneous vasculitis, more diffuse extra-glandular manifestations and serum markers of monoclonal B-cell proliferation (19). Cryoglobulinemia, in particular, has been associated with PNS ESSDAI (20) and all these markers appear to be predictive of SMP damage in pSS (14, 15, 17, 21). These observations can also explain the higher need of GCs and IS therapy in patients with SMP and reinforce the hypothesis that SMP and pure sensory neuropathy patient subgroups may belong to a distinct disease phenotype which may reflect the existence of different pathogenic mechanisms underlying the high variability of disease-specific clinical and immunological features with consequent variable prognosis. Altogether, the results of our study may suggest that pSS patients with pure sensory neuropathy show an intermediate degree of disease severity between subjects with SMP and those with no PNS involvement.

In this setting, it has been long evident that anti-Ro/SSA and anti-La/SSB autoantibodies are associated with more diffuse glandular infiltrate and several extra-glandular manifestations, but not with PNS involvement (22). In fact, a recent analysis of the Big Data Sjögren Project Consortium international registry showed that anti-Ro/SSA and anti-La/SSB positive patients were characterized by higher frequency of activity in many ESSDAI domains except for PNS and CNS ones (18). Similarly, in a wide prospective Korean study, anti-Ro/SSA-negative patients were characterized by higher prevalence of PNS ESSDAI domain features in comparison to anti-Ro/SSA-positive subjects (23).

We confirmed a relatively high prevalence of pure sensory neuropathy (14–16, 21, 24–36). This manifestation may have a negative impact on disease outcome and patient quality of life due to disabling symptoms, often requiring immunosuppressive therapies (37–39). pSS subjects with evidence of pure sensory neuropathies, including SFN and DRG, are usually characterized by a low prevalence of serological markers of chronic B-cell activation, including ANA, anti-Ro/SSA, anti-La/SSB and rheumatoid factor (14–16, 21, 24–36). The reason why we failed to confirm this observation is probably due to the low proportion of “seronegative” PSS patients and of patients – around 10% - who had the neurological diagnosis before pSS diagnosis in our cohort. In fact, pure sensory neuropathy can be characterized by sicca symptoms *per se*, which may represent a possible confounding factor for the diagnosis of pSS (33). Thus, the unusual high proportion of “seronegative” pSS patients with pure sensory neuropathy reported in several studies may question the accuracy of pSS diagnosis. Our findings support the idea that pure sensory neuropathy developing in the course of pSS represents a distinct entity and is probably sustained by different pathogenic mechanisms compared to pure sensory neuropathy complicated by sicca syndrome, often erroneously diagnosed as “seronegative” pSS.

Our data confirm the hypothesis that SMP is mainly sustained by vasculitis and immune-complex deposition disease, while pure sensory neuropathy may be caused by a direct immune-mediated damage (19). This aspect also reflects on the treatment of the disease. In fact, although immunosuppressive therapy was more prevalent in both our cohorts with PNS involvement, compared to subjects with no PNS disease, the recently published EULAR recommendations suggest first-line immunosuppressive therapy in case of SMP and DRG and symptomatic therapy in patients with axonal sensory polyneuropathy (40).

In this context, it is intriguing to analyze these data from a neurological perspective. In fact, the most common immune-mediated causes of SFN and DRG are represented by pSS and celiac disease (41–43). Since PNS manifestations, in particular SFN and DRG, represent typical clinical features of celiac disease which, in turn, has been recently shown to be closely associated with pSS (44), an intriguing and still unexplored common pathogenic network may be hypothesized.

We believe the main strengths of our study are the homogeneous characterization of the variables analyzed and the choice to only identify patients with symptomatic PNS involvement. This approach may lead to a formal underestimation of PNS involvement, including subjects with subclinical PNS alterations identified only by routine screening of all pSS patients with nerve conduction studies. However, this latter approach would have a questionable rationale in a real-life setting.

Apart from its retrospective nature, one of the main limitations of our study is that the low number of patients with PNS involvement, in particular the group with pure sensory neuropathy including only 25 subjects, may mask the presence of other significant differences between the groups analyzed. However, it is unlikely that the overall findings and interpretation would be significantly contradicted by a similar but larger cohort. Additionally, because this is a cohort study, we cannot exclude that in a proportion of subjects with pure sensory neuropathy, the disease may have subsequently evolved into a SMP and that some of those with SMP may have had a pure sensory neuropathy at onset, with a likely compensatory effect on the results.

In conclusion, the present study suggests that PNS involvement is a rather rare but prognostically relevant manifestation in pSS patients. The most frequent types of PNS involvement in pSS were SMP and pure sensory neuropathy. The significantly different immunological and clinical features between the two groups likely reflect peculiar distinct pathogenic mechanisms implicated. An accurate diagnosis of the type of neurologic involvement in pSS is needed to stratify patients and optimize the choice of the most appropriate treatment according to the underlying pathogenic mechanism. This is of particular importance considering the prognostic relevance of neurological involvement in these patients and the different therapeutic approaches commonly employed to treat the single NS manifestations.

## Data Availability Statement

The raw data supporting the conclusions of this article will be made available by the authors, without undue reservation.

## Ethics Statement

The studies involving human participants were reviewed and approved by Comitato Etico Regionale Umbria. Written informed consent from the participants’ legal guardian/next of kin was not required to participate in this study in accordance with the national legislation and the institutional requirements.

## Author Contributions

GC, RGe and EB contributed to conception and design of the study. GC performed the statistical analysis. GC, RGe and EB wrote the first draft of the manuscript. CP wrote sections of the manuscript. All authors contributed to the article and approved the submitted version.

## Conflict of Interest

The authors declare that the research was conducted in the absence of any commercial or financial relationships that could be construed as a potential conflict of interest.
